# Associations of Depression, Antidepressants with Atrial Fibrillation Risk in HFpEF Patients

**DOI:** 10.31083/j.rcm2510370

**Published:** 2024-10-22

**Authors:** Yonghui Fu, Shenghui Feng, Zhenbang Gu, Xiao Liu, Wengen Zhu, Bo Wei, Linjuan Guo

**Affiliations:** ^1^Department of Psychiatry, Jiangxi Mental Hospital, 330029 Nanchang, Jiangxi, China; ^2^Medical Department, Queen Mary School, Nanchang University, 330006 Nanchang, Jiangxi, China; ^3^Department of Cardiology, The First Affiliated Hospital of Sun Yat-Sen University, 510080 Guangzhou, Guangdong, China; ^4^Department of Cardiology, Sun Yat-sen Memorial Hospital of Sun Yat-sen University, 510030 Guangzhou, Guangdong, China; ^5^Department of Cardiology, Jiangxi Provincial People's Hospital, The First Affiliated Hospital of Nanchang Medical College, 330006 Nanchang, Jiangxi, China

**Keywords:** HFpEF, depression, antidepressants, atrial fibrillation, outcome

## Abstract

**Background:**

Studies dedicated to exploring the incidence of atrial fibrillation (AF) in patients with concurrent depression and heart failure with preserved ejection fraction (HFpEF) are scarce. The impact of antidepressant therapy on AF risk within this population remains unclear. Our current study aimed to investigate the link between depression and AF risk in HFpEF patients and to assess the influence of antidepressant medication on the development of AF.

**Methods:**

We utilized Kaplan-Meier estimates to determine the event-free status for AF and applied the Log-rank test for comparative analysis between groups. The associations were quantified using univariate and multivariate Cox proportional hazards regression models, with results expressed as hazard ratios (HR) and 95% confidence intervals (CI).

**Results:**

Among the 784 patients in the Treatment of Preserved Cardiac Function Heart Failure with an Aldosterone Antagonist (TOPCAT) trial, 29.1% (228) were identified with major depression. After adjusting for significant confounders, compared with mild depression, major depression at baseline was not linked to the incidence of AF (adjusted HR = 0.82, 95% CI: 0.46–1.49). Additionally, compared with controls, antidepressant use at baseline did not significantly influence the risk of incident AF in patients with HFpEF and major depression (adjusted HR = 0.41, 95% CI: 0.08–2.10).

**Conclusions:**

The presence of major depression at baseline did not elevate the risk of incident AF among individuals with HFpEF. Additionally, the use of antidepressants showed no correlation with an increased rate of AF among HFpEF patients with comorbid major depression.

**Clinical Trial Registration:**

URL: https://clinicaltrials.gov/study/NCT00094302. Unique identifier: NCT00094302.

## 1. Introduction

Heart failure with preserved ejection fraction (HFpEF) represents the predominant form of heart failure, accounting for over 50% of cases [1]. The incidence of HFpEF has surged alongside an aging population and the rising prevalence of conditions such as diabetes, obesity, and metabolic syndrome [2, 3]. Despite its high morbidity and mortality rates, there are limited effective treatment options for HFpEF. Most current therapies approved for heart failure with reduced ejection fraction (HFrEF) have not demonstrated efficacy in managing HFpEF.

Depression, characterized by a spectrum of psychiatric and physical symptoms such as enduring sadness, diminished interest, exhaustion, pain, and disrupted sleep, is a mood disorder with significant implications for health. Research has increasingly highlighted its potential links to cardiovascular diseases (e.g., coronary artery disease [4]), and its influence as a risk factor for both mortality and morbidity in HF patients [5, 6]. Shared mechanisms, such as the hyperactivation of the hypothalamic-pituitary-adrenal (HPA) axis and the disruption of inflammatory pathways involving cytokines like C-reactive protein, interleukin-6, interleukin-10, and tumor necrosis factor-alpha, have been posited to explain the high comorbidity rate between depression and cardiovascular diseases. These inflammatory mediators can disrupt the heart’s electrophysiology [7, 8]. However, the majority of research has focused on HFrEF, with comparatively less data available for HFpEF.

Atrial fibrillation (AF), the most common arrhythmia, is associated with increased risks of stroke and mortality. AF and HFpEF are often referred to as “vicious twins” [9]. They are closely interrelated and contribute to adverse cardiovascular outcomes [10]. HF is recognized as a significant risk factor for the development of new-onset AF [11], with HFpEF patients exhibiting a prevalence of AF ranging from 15% to 41%. The interplay between depression, antidepressant medication, and AF is an area of growing investigation [12]. Given the recognized links between HFpEF, depression, and the risk of AF, our study undertook a retrospective analysis of data from the Treatment of Preserved Cardiac Function Heart Failure with an Aldosterone Antagonist (TOPCAT) trial [13] to explore the relationship between major depression at baseline and the subsequent development of AF in HFpEF patients. Additionally, we examined the potential impact of antidepressant use on the emergence of new-onset AF in individuals with comorbid depression and HFpEF.

## 2. Materials and Methods

### 2.1 Study Population

The TOPCAT trial was a comprehensive, randomized, placebo-controlled study that assessed the impact of spironolactone, an aldosterone antagonist, on patients with HFpEF. A thorough depiction of the study’s design and data collection protocols has been documented [13]. Approval from ethics committees was secured at each participating site, with all participants providing informed consent. The study’s primary goal was to determine the efficacy of spironolactone in improving a composite endpoint that encompasses cardiovascular mortality, aborted cardiac arrest, or hospitalization due to heart failure in the HFpEF patient cohort.

The TOPCAT trial included 3445 individuals at 233 centres from the United States, Canada, Russia, and Georgia, with eligibility criteria focusing on symptomatic heart failure patients exhibiting a left ventricular ejection fraction of ≥45%, and either a history of heart failure hospitalization within the last 12 months or elevated natriuretic peptide levels (B-type natriuretic peptide (BNP) ≥100 pg/mL or N-terminal pro B-type natriuretic peptide (NT-proBNP) ≥360 pg/mL) within 60 days prior to the study. Participants were required to be aged 50 or above, with controlled systolic blood pressure (<140 mmHg or ≤160 mmHg if on antihypertensive medication), and a serum potassium level below 5.0 mmol/L. Exclusions were based on life expectancy, severe renal impairment (estimated glomerular filtration rate (e-GFR) <30 mL/min/1.73 m^2^ or serum creatinine ≥2.5 mg/dL), and other specific conditions or medications [13, 14].

**Fig. 1.**
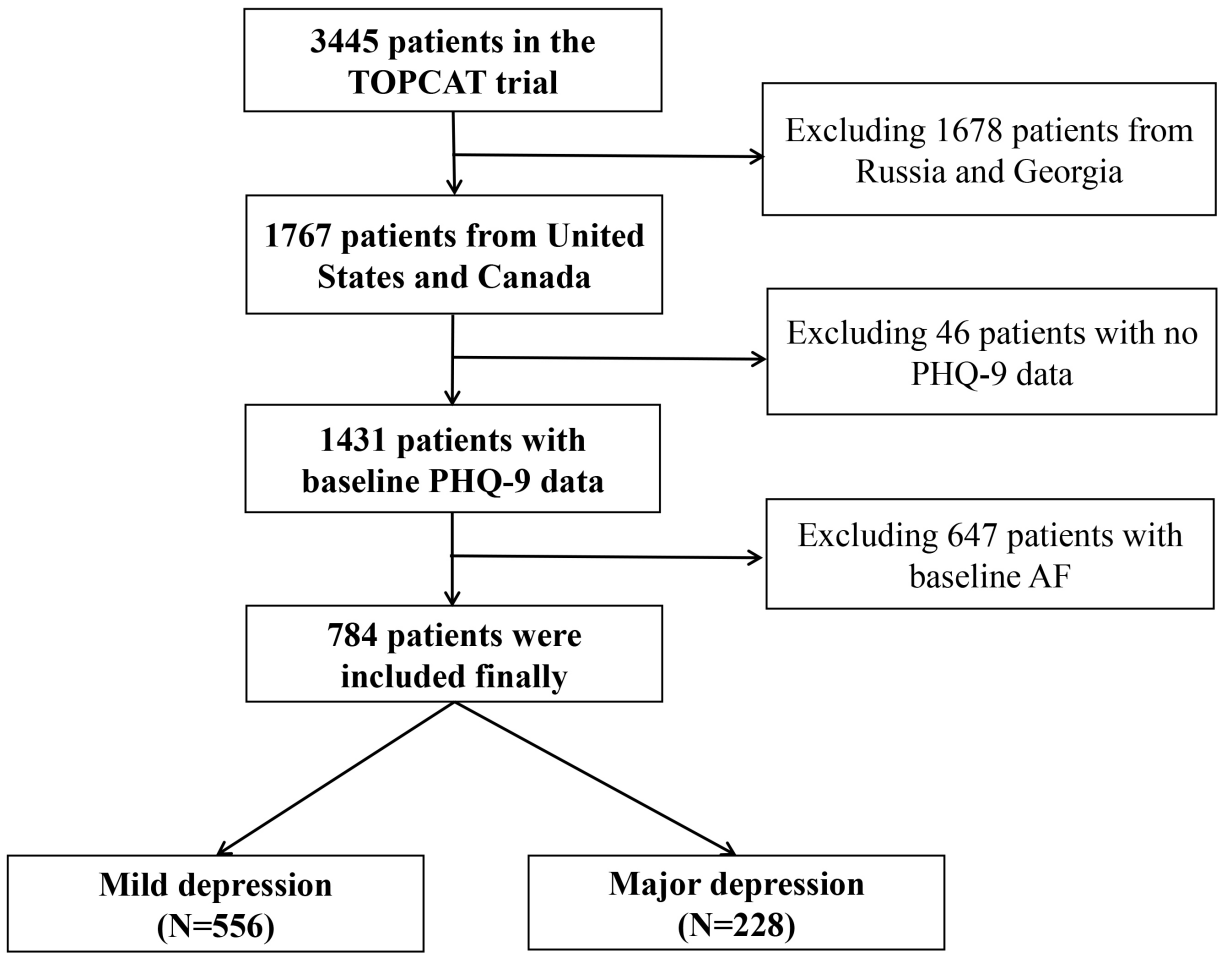
**The detailed inclusion and exclusion flow chart to clarify participant selection for our analysis**. TOPCAT, Treatment of Preserved Cardiac Function Heart Failure with an Aldosterone Antagonist Trial; PHQ-9, Patient Health Questionnaire-9; AF, atrial fibrillation.

For our analysis, 784 patients were finally included (Fig. 1). Our methodology adhered to the STROBE (Strengthening the Reporting of Observational Studies in Epidemiology) guidelines for reporting.

### 2.2 Diagnosis of Major Depression and Antidepressant Medication Ascertainment

Depression assessment within the TOPCAT trial utilized the Patient Health Questionnaire-9 (PHQ-9), a validated instrument for evaluating the presence and severity of depression symptoms over the prior two weeks. Scoring is based on a scale from ‘0’ (not at all) to ‘3’ (nearly every day), with scores ≥10 indicating major depression (i.e., clinical depression) with a reported sensitivity and specificity of 88% [15]. A PHQ-9 score of less than 10 points indicated mild depression (i.e., non-clinical depression). The PHQ-9 scores were categorized for analysis, and baseline medication data, including antidepressant use, was compiled. Antidepressant classification was referenced against the World Health Organization-Drug Dictionary.

### 2.3 Follow-up and AF Ascertainment

Participant follow-up occurred quarterly for the first year, and every six months thereafter, with incidence of AF events confirmed via 12-lead electrocardiograph (ECG) or documented medical records per predefined criteria by the clinical endpoint committee at Brigham and Women’s Hospital [14].

### 2.4 Data Analysis

Baseline characteristics were presented using means and standard deviations for normally distributed continuous variables and medians with interquartile ranges for others. Unpaired *T*-tests and Pearson χ^2^ tests compared group characteristics. AF event-free survival was estimated using Kaplan-Meier methods, with Log-rank tests for group comparisons. Cox proportional regression models were applied to determine the association of AF with depression and antidepressants, reported as hazard ratios (HR) and 95% confidence intervals (CI). Multivariate analysis included adjustments for various demographic, clinical, and treatment-related factors, including age, sex, New York Heart Association (NYHA) class, body mass index, current smoking, previous hospitalization for HF, previous myocardial infarction, previous stroke, hypertension, peripheral vascular disease, diabetes mellitus, percutaneous coronary intervention, chronic obstructive pulmonary disease, angiotensin-converting enzyme inhibitor (ACEI), angiotensin receptor blocker (ARB), diuretic, estimated-glomerular filtration rate, previous coronary artery bypass grafting, anti-antidepressant (except for the analysis of antidepressant), and randomization of spironolactone. Sensitivity analyses employed inverse probability of treatment weighting (IPTW) [14] and propensity score matching to ensure the comparability of groups. Statistical analyses were conducted using SPSS Statistics (26.0, IBM Corp, Armonk, NY, USA), Stata/MP (16.0, Stata Corp LLC, College Station, TX, USA), and R (4.1.3, R core team, Vienna, Austria), with a *p*-value ≤ 0.05 considered statistically significant.

## 3. Results 

### 3.1 Baseline Characteristics of Participants

Table 1 outlines the baseline characteristics of our study population categorized by the extent of depression. A total of 784 patients were enrolled, of which 29.1% (n = 228) were diagnosed with major depression based on their PHQ scores. Compared to those with mild depression, patients with HFpEF and major depression were notably younger and had a higher body mass index compared to those with mild depression (*p* < 0.05). Additionally, this group exhibited a worse NYHA functional classification, a higher prevalence of diabetes mellitus and chronic obstructive pulmonary disease, and more frequent use of ACEI/ARBs and diuretics (all *p* < 0.05).

**Table 1.** **Characteristics of HFpEF patients according to baseline depression status**.

		Mild depression	Major depression	*p*-value
		N = 556	N = 228	
Spironolactone randomization		282 (50.72%)	110 (48.25%)	0.529
Demographic				
	Age (years)	71.0 (63.0–79.0)	64.0 (58.0–74.0)	<0.001
	Heart rate, bpm	68 (11)	70 (11)	0.077
	SBP, mmHg	69 (60–78)	71 (62–80)	0.053
	DBP, mmHg	130 (118–140)	130 (117–141)	0.618
	BMI, kg/m^2^	34.2 (8.5)	37.2 (9.1)	<0.001
	Sex, male	284 (51.08%)	108 (47.37%)	0.345
	Current smoking	45 (8.09%)	25 (10.96%)	0.200
	NT pro-BNP^*^ (pg/mL)	813 (485–1641)	900 (494–1748)	0.682
	e-GFR (mL/(min/1.73 m^2^))	65.09 (22.53)	66.51 (25.19)	0.439
NYHA				<0.001
	I–II	367 (66.01%)	118 (51.98%)	
	III–IV	189 (33.99%)	109 (48.02%)	
Chronic health conditions, n%				
	Previous CHF	303 (54.50%)	140 (61.40%)	0.076
	Previous myocardial infarction	147 (26.44%)	47 (20.61%)	0.086
	Previous stroke	50 (8.99%)	22 (9.65%)	0.773
	Previous PCI	153 (27.52%)	50 (21.93%)	0.105
	Previous CABG	131 (23.56%)	55 (24.12%)	0.867
	COPD	91 (16.37%)	52 (22.81%)	0.034
	Hypertension	507 (91.19%)	212 (92.98%)	0.408
	Peripheral vascular disease	85 (15.29%)	38 (16.67%)	0.632
	Diabetes mellitus	285 (51.26%)	147 (64.47%)	<0.001
Medication				
	ACEI/ARB	422 (75.90%)	189 (82.89%)	0.032
	Beta-blocker	460 (82.73%)	186 (81.58%)	0.700
	Calcium channel blocker	220 (39.57%)	102 (44.74%)	0.182
	Diuretic	478 (85.97%)	210 (92.11%)	0.017
	Statin	403 (72.48%)	170 (74.56%)	0.551
	Warfarin	42 (7.55%)	17 (7.46%)	0.962
	Aspirin	400 (71.94%)	152 (66.67%)	0.142

HFpEF, heart failure with preserved ejection fraction; BMI, body mass index; NT pro-BNP, N-terminal pro B-type natriuretic peptide; NYHA, New York Heart Association; bpm, beat per minutes; SBP, systolic blood pressure; DBP, diastolic blood pressure; e-GFR, estimated glomerular filtration rate; CHF, chronic heart failure; CABG, coronary artery bypass grafting; PCI, percutaneous coronary intervention; COPD, chronic obstructive pulmonary disease; ACEI, angiotensin converting enzyme inhibitor; ARB, angiotensin receptor blocker. *Available in 147 patients.

### 3.2 Association of Incident AF in HFpEF Patients with Major Depression at Baseline

**Fig. 2.**
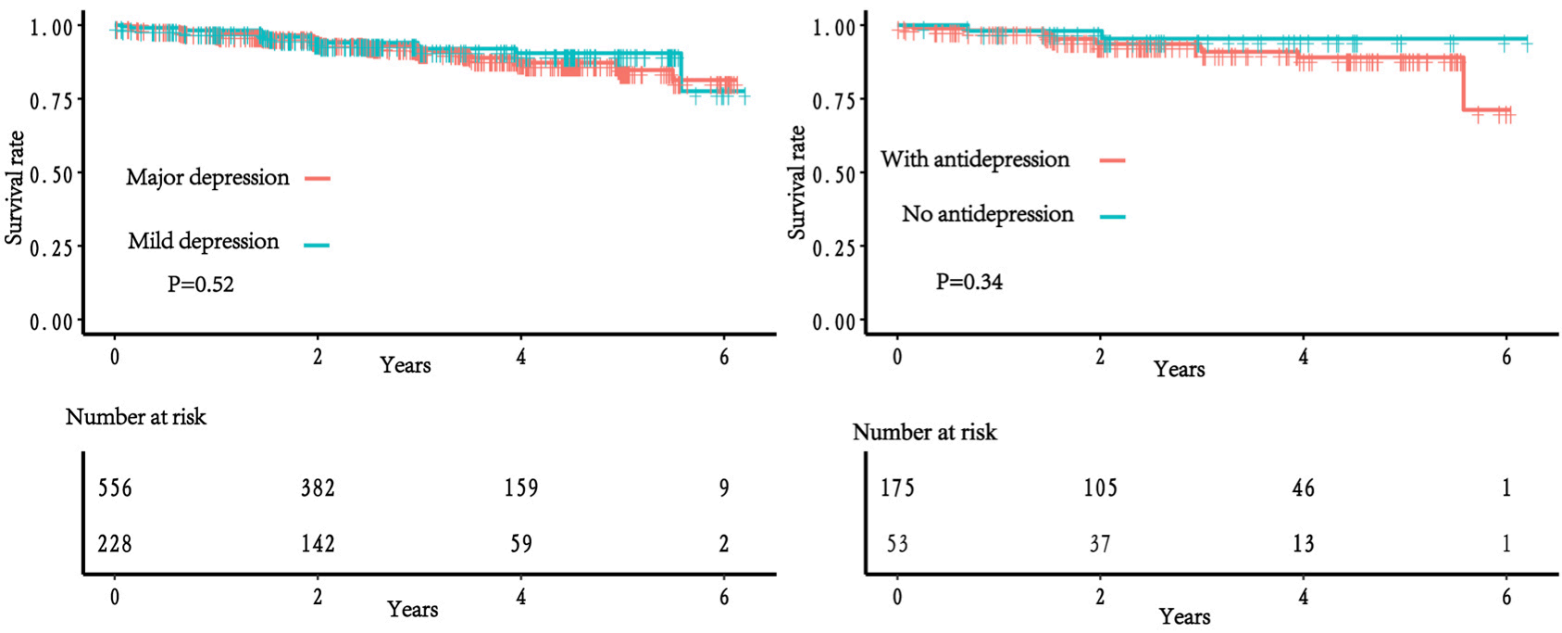
**Kaplan-Meier curves for atrial fibrillation incidence among patients with heart failure with preserved ejection fraction**. Left panel: according to baseline depressive state. Right panel: according to anti-depression treatments with major depression.

Table 2 details the incidence rates and hazard ratios for AF in relation to major depression at baseline. The Kaplan-Meier survival curves, depicted in Fig. 2, illustrate the clinical outcomes. In the unadjusted model, the incident AF rates were 2.36 and 2.89 per 100 person-years for patients with and without major depression, respectively, showing no significant difference in risk (HR = 0.82; 95% CI: 0.46–1.46, *p* = 0.505).

**Table 2.** **Association of depression with AF incidence in patients with HFpEF**.

	Cases/N	Person-years	Incidence rate, per 100 person-year	Crude HR (95% CI)	Adjusted HR* (95% CI)	IPTW
				*p*-value	*p*-value	*p*-value
Mild depression	48/556	1657.7	2.89	Ref	Ref	Ref
Major depression	15/228	636.7	2.36	0.82 (0.46, 1.46)	0.82 (0.46, 1.49)	0.85 (0.46, 1.57)
				0.505	0.518	0.598

*Adjusted for age, sex, NYHA class; BMI; current smoking; previous hospitalization for CHF; previous myocardial infarction; previous stroke; hypertension; peripheral vascular disease; diabetes mellitus; ACEI/ARB; diuretic, anti-depression; eGFR; COPD, previous PCI, previous CABG, randomization. HFpEF, heart failure with preserved ejection fraction; HR, hazard ratios; CI, confidence intervals; NYHA, New York Heart Association; AF, atrial fibrillation; e-GFR, estimated glomerular filtration rate; ACEI, angiotensin converting enzyme inhibitor; ARB, angiotensin receptor blocker; COPD, chronic obstructive pulmonary disease; IPTW, inverse probability of treatment weighting; BMI, body mass index; CHF, chronic heart failure; CABG, coronary artery bypass grafting; PCI, percutaneous coronary intervention.

After adjusting for significant confounders, compared with mild depression, major depression at baseline was not linked to the incidence of AF (adjusted HR = 0.82; 95% CI: 0.46–1.49, *p* = 0.518). Further adjustment using IPTW confirmed these findings (HR = 0.85; 95% CI: 0.46–1.57, *p* = 0.598).

### 3.3 Effect of Anti-depression Treatment on Incident AF in Patients with HFpEF

An ancillary analysis focused on the relationship between antidepressant treatment and incident AF in patients with comorbid HFpEF and major depression. Table 3 presents the baseline characteristics of these patients (N = 228), with 53 (23.2%) receiving antidepressant treatment at baseline. Notably, patients on antidepressants had higher systolic blood pressure and smoking rates.

**Table 3.** **Characteristics of HFpEF patients according to anti-depression treatments with major depression**.

		No antidepressants	Antidepressants	*p*-value
		(N = 175)	(N = 53)	
Spironolactone randomization		80 (45.71%)	30 (56.60%)	0.165
Demographic				
	Age (years)	64.0 (58.0–74.0)	64.0 (58.0–69.0)	0.629
	Sex, male	84 (48.00%)	24 (45.28%)	0.729
	BMI, kg/m^2^	37.27 (9.38)	36.78 (8.35)	0.728
	Heart rate, bpm	69 (11)	71 (12)	0.371
	SBP, mmHg	129 (116–139)	138 (123–149)	0.013
	DBP, mmHg	70 (60–79)	72 (66–80)	0.062
	e-GFR (mL/(min*1.73 m^2^))	59.85 (47.67–81.34)	65.38 (51.27–80.56)	0.599
	Current smoking	14 (8.00%)	11 (20.75%)	0.009
	NYHA	-	-	0.649
	I–II	89 (51.15%)	29 (54.72%)	-
	III–IV	85 (48.85%)	24 (45.28%)	-
Chronic health conditions, n%				
	Previous CHF	107 (61.14%)	33 (62.26%)	0.883
	Previous myocardial infarction	40 (22.86%)	7 (13.21%)	0.128
	Previous stroke	18 (10.29%)	4 (7.55%)	0.554
	Previous CABG	45 (25.71%)	10 (18.87%)	0.307
	Previous PCI	42 (24.00%)	8 (15.09%)	0.170
	COPD	38 (21.71%)	14 (26.42%)	0.475
	Hypertension	162 (92.57%)	50 (94.34%)	0.659
	Peripheral vascular disease	31 (17.71%)	7 (13.21%)	0.441
	Diabetes mellitus	112 (64.00%)	35 (66.04%)	0.786
Medication				
	ACEI/ARB	144 (82.29%)	45 (84.91%)	0.657
	Beta-blocker	144 (82.29%)	42 (79.25%)	0.617
	Calcium channel blocker	76 (43.43%)	26 (49.06%)	0.470
	Diuretic	160 (91.43%)	50 (94.34%)	0.491
	Aspirin	113 (64.57%)	39 (73.58%)	0.223
	Warfarin	14 (8.00%)	3 (5.66%)	0.570

*Adjusted for age, sex, NYHA class; BMI; current smoking, previous hospitalization for CHF; previous myocardial infarction; previous stroke; hypertension; peripheral vascular disease; diabetes mellitus; ACEI/ARB; diuretic; e-GFR; COPD; previous PCI, previous CABG, spironolactone randomization. HFpEF, heart failure with preserved ejection fraction; BMI, body mass index; SBP, systolic blood pressure; DBP, diastolic blood pressure; CHF, chronic heart failure; CABG, coronary artery bypass grafting; PCI, percutaneous coronary intervention; COPD, chronic obstructive pulmonary disease; ACEI, angiotensin converting enzyme inhibitor; ARB, angiotensin receptor blocker; NYHA, New York Heart Association; e-GFR, estimated glomerular filtration rate.

Table 4 and Fig. 2 show the event rates and HRs for patients with and without antidepressant treatment. In both unadjusted and adjusted analyses, compared with controls, antidepressant use at baseline did not significantly influence the risk of AF in patients with HFpEF and major depression (crude HR = 0.46; 95% CI: 0.10–2.06, *p* = 0.313; adjusted HR = 0.41; 95% CI: 0.08–2.10, *p* = 0.285). In addition, further adjustment using IPTW confirmed these findings (HR = 0.46; 95% CI: 0.10–2.12, *p* = 0.316).

**Table 4.** **Association of anti-depression treatments with AF incidence in patients with HFpEF**.

	Cases/N	Person-years	Incidence rate, per 100 person-year	Crude HR (95% CI)	Adjusted HR* (95% CI)	IPTW
				*p*-value	*p*-value	*p*-value
No antidepression	13/175	484.1	2.6	Ref	Ref	Ref
With antidepression	2/53	152.6	1.3	0.46 (0.10, 2.06)	0.41 (0.08, 2.10)	0.46 (0.10, 2.12)
				0.313	0.285	0.316

*Adjusted for age, sex, NYHA class; BMI; previous hospitalization for CHF; previous myocardial infarction; previous stroke; hypertension; peripheral vascular disease; diabetes mellitus; current smoking, ACEI/ARB; diuretic, e-GFR, COPD; previous PCI, previous CABG, randomization. HR, hazard ratios; CI, confidence intervals; AF, atrial fibrillation; NYHA, New York Heart Association; e-GFR, estimated glomerular filtration rate; ACEI, angiotensin converting enzyme inhibitor; ARB, angiotensin receptor blocker; COPD, chronic obstructive pulmonary disease; IPTW, inverse probability of treatment weighting; HFpEF, failure with preserved ejection fraction; BMI, body mass index; CHF, chronic heart failure; CABG, coronary artery bypass grafting; PCI, percutaneous coronary intervention.

## 4. Discussion

In the TOPCAT study cohort, 29.1% of the participants were diagnosed with major depression, and of this group, 23.2% received antidepressant treatment. Our study’s findings indicate that the presence of comorbid major depression in patients with HFpEF did not increase the risk of developing AF when compared to those with mild depression. Moreover, the initiation of antidepressant therapy at baseline did not appear to influence the incidence rate of AF within this patient group.

Previous investigations have delved into the relationship between comorbid depression and the adverse outcomes of HF [16]. In the broader HF population, depression is considered a harbinger of poor prognosis, linked to increased mortality, cardiac events, hospital readmissions, and heightened demand for healthcare services [17, 18, 19]. AF in HF patients has also been correlated with heightened mortality risks and unfavorable outcomes [20]. Studies have shown an association between HF, depression, and the risk of new-onset AF [10, 11, 21, 22]. The increased risk of AF in the context of depression is hypothesized to stem from systemic inflammatory responses and the dysregulation of the HPA axis, resulting in myocardial infiltration by inflammatory cells [11]. Additionally, several potential mechanisms underpin the strong correlation between AF and HF. Notably, the enlargement of the left atrium and the associated atrial fibrosis are recognized as significant proarrhythmic factors in HF patients [23]. The irregular distribution of gap junctions, the loss of cell-to-cell coupling within fibrotic regions, and disruptions in ion channel regulation are all implicated in the electrical remodeling of the heart. In the context of atrial remodeling, there is a reduction in the L-type calcium current, the transient outward potassium current, and the slow delayed rectifier potassium current within atrial myocytes [24]. Conversely, an increase in the transient inward sodium-calcium exchanger current can trigger delayed afterdepolarizations, which may precipitate arrhythmias, including AF [25, 26]. The role of gap junctions, involving atrial connexin proteins, in creating impulse propagation inhomogeneity, also contributes to the formation of re-entry circuits that are prone to AF [27]. However, most of the aforementioned mechanisms linking AF with HF are derived from studies involving patients with HFrEF. It has been suggested that conventional pharmacotherapies do not confer benefits in depressed HFrEF patients [28]. The applicability of these mechanisms to patients with HFpEF is yet to be determined and warrants further investigation.

The significant differences between HFpEF and HFrEF have been identified in previous studies. In the chronic outpatient setting, the filling pressures of LV were higher in patients with HFrEF compared to those with HFpEF [29]. In addition, compared with the HFpEF group, the left atrial global longitudinal strain of HFrEF patients was profoundly lower [29]. At present, the number of studies focusing on HFpEF is relatively limited, which restricts the persuasion of these theories applied to the HFpEF group. Considering the distinct differences between HFpEF and HFrEF, patients with HFpEF deserve to be studied independently. Many clinical outcomes in HFpEF patients with depression at baseline have shown distinctions compared with previous HFrEF patients: major depression at baseline did not increase the rate of mortality or rehospitalization in the HFpEF group [30]. These results suggest that the occurrence and risk of other clinical events in the HFpEF population might also be different from what was previously thought.

Our study yields surprising findings regarding the interplay among HFpEF, depression, and the risk of developing AF. Theoretical frameworks suggest that depression, AF, and HFpEF are linked through a shared mechanism of systemic inflammation. A novel perspective posits that HFpEF is an inflammatory disorder, and when it coexists with depression—characterized by heightened levels of proinflammatory cytokines—it may precipitate AF-related symptoms such as endothelial dysfunction, oxidative stress, and microvascular inflammation more readily [31, 32]. Nevertheless, conflicting theories persist. Other recognized risk factors for HF and AF, including tachycardia or cardiomyopathy induced by irregularity, with implications for hemodynamics, structure, cellular function, and neurohormonal activity [33, 34], have been primarily associated with HFrEF. Their relevance to HFpEF is not well understood and merits further investigation.

Moreover, the impact of antidepressant treatment on the risk of AF has been a contentious issue. Within the spectrum of antidepressants, tricyclic antidepressants are known for their adverse effects on cardiac conduction and potential cardiotoxicity [35]. Elevated serum serotonin levels, associated with the use of selective serotonin reuptake inhibitors, may lead to calcium overload, focal atrial extrasystoles, and an increased risk of AF [36, 37]. The use of antidepressants could also result in prolonged QTc intervals, potentially inducing arrhythmias [38]. Conversely, several studies propose that antidepressants might modulate the imbalance of inflammatory cytokines caused by depression [39, 40, 41], which could mitigate the risk of AF induced by systemic inflammation and abnormal cardiovascular metabolism.

The dual nature of these mechanisms may elucidate our findings. In conjunction with the findings by Liu *et al*. [30], our results imply that among patients with HFpEF and major depression at baseline, there may be less cause for concern regarding the use of antidepressants leading to adverse cardiovascular outcomes, including AF. Standard antidepressant therapy appears to be viable for this population. Future trials are warranted to substantiate our findings.

### Study Implications

Our current study presents unexpected insights into the relationship between HFpEF, depression, and the risk of AF. While depression, AF, and HFpEF are theorized to share systemic inflammation mechanisms, our results indicated that the coexistence of HFpEF with depression did not necessarily trigger AF-related symptoms as easily as previously thought. Furthermore, the impact of antidepressant treatment on the risk of incident AF has been a subject of debate. While some antidepressants might affect cardiac conduction and cause cardiotoxicity, others could potentially mitigate the risk of inflammation-induced cardiovascular abnormalities and new-onset AF by modulating inflammatory cytokines. These insights underscore the complexity of treating HFpEF patients with comorbid depression and highlight the need for a nuanced approach to clinical decision-making. Our findings advocate for further research and careful consideration when prescribing antidepressants to this patient population, taking into account both the potential risks and benefits in the context of their cardiovascular health.

## 5. Limitations

Several limitations should be noted in our study. First, we recognize the constraint of relying solely on the PHQ-9 to measure depressive symptoms in HFpEF patients. Given the symptomatic overlap between depression and HF, effective management of HF symptoms could lead to improvements in depressive symptoms. Furthermore, the self-reported nature of depression-induced symptoms and the potential for over-diagnosis are concerns. The PHQ-9, primarily validated for use in the United States and Canada, may not be fully applicable to our international cohort of HFpEF patients, possibly affecting the accuracy of outcomes related to depression at baseline. Second, we concede the possibility of residual confounding factors, such as the absence of specific anti-arrhythmic medications like amiodarone and flecainide, which could influence the findings. Third, our study did not specifically assess the dosages and types of antidepressants administered. The impact of these factors, along with the severity of depression and demographic variables, on the risk of new-onset AF in the HFpEF population with baseline depression warrants further investigation. Finally, the lack of continuous monitoring methods, including Holter monitoring, implantable loop recorders, or transtelephonic monitoring systems, may have resulted in an underestimation of the AF incidence rate.

## 6. Conclusions 

According to the findings from the TOPCAT trial, the presence of major depression at baseline did not elevate the likelihood of developing AF among individuals with HFpEF. Additionally, the utilization of antidepressant medications showed no correlation with a heightened rate of AF within patients with comorbid major depression and HFpEF. Further study could confirm our findings.

## Availability of Data and Materials

Data will be made available on request.
